# Detection of Myocardial Infarction Using ECG and Multi-Scale Feature Concatenate

**DOI:** 10.3390/s21051906

**Published:** 2021-03-09

**Authors:** Jia-Zheng Jian, Tzong-Rong Ger, Han-Hua Lai, Chi-Ming Ku, Chiung-An Chen, Patricia Angela R. Abu, Shih-Lun Chen

**Affiliations:** 1 Department of Biomedical Engineering, Chung Yuan Christian University, Taoyuan City 320314, Taiwan; g10875014@cycu.edu.tw (J.-Z.J.); g10975008@cycu.edu.tw (H.-H.L.); g10575014@cycu.edu.tw (C.-M.K.); 2 Department of Electrical Engineering, Ming Chi University of Technology, New Taipei City 243303, Taiwan; 3 Department of Information Systems and Computer Science, Ateneo de Manila University, Quezon City 1108, Philippines; pabu@ateneo.edu; 4 Department of Electronic Engineering, Chung Yuan Christian University, Taoyuan City 320314, Taiwan; chrischen@cycu.edu.tw

**Keywords:** accuracy, convolution neural network (CNN), classifiers, electrocardiography, k-fold validation, myocardial infarction, sensitivity

## Abstract

Diverse computer-aided diagnosis systems based on convolutional neural networks were applied to automate the detection of myocardial infarction (MI) found in electrocardiogram (ECG) for early diagnosis and prevention. However, issues, particularly overfitting and underfitting, were not being taken into account. In other words, it is unclear whether the network structure is too simple or complex. Toward this end, the proposed models were developed by starting with the simplest structure: a multi-lead features-concatenate narrow network (N-Net) in which only two convolutional layers were included in each lead branch. Additionally, multi-scale features-concatenate networks (MSN-Net) were also implemented where larger features were being extracted through pooling the signals. The best structure was obtained via tuning both the number of filters in the convolutional layers and the number of inputting signal scales. As a result, the N-Net reached a 95.76% accuracy in the MI detection task, whereas the MSN-Net reached an accuracy of 61.82% in the MI locating task. Both networks give a higher average accuracy and a significant difference of *p* < 0.001 evaluated by the *U* test compared with the state-of-the-art. The models are also smaller in size thus are suitable to fit in wearable devices for offline monitoring. In conclusion, testing throughout the simple and complex network structure is indispensable. However, the way of dealing with the class imbalance problem and the quality of the extracted features are yet to be discussed.

## 1. Introduction

Myocardial infarction (MI), defined in pathology as myocardial cell death due to prolonged ischemia, is a serious heart disease that can cause death and disability [1]. According to the American Heart Association, it is estimated that 750,000 Americans have a heart attack every year, with approximately 116,000 deaths [2]. Therefore, early diagnosis and detection are the utmost important task. Nowadays, several methods already exist to recognize MI, including electrocardiogram (ECG), biomarkers, imaging technique, or defined by pathology. Yet, the non-invasive ECG is the most economical and widely used one for the sake of immediate treatment strategies among them [3,4,5,6,7]. Performing ECG analysis manually may not merely be time-consuming but leads to inter-observer variability [8,9]. Consequently, a computer-aided diagnosis system may come in handy to solve these difficulties. Moreover, wearable devices are common technology in recent days and are rising in numbers. Wang et al. [10] provided a wearable ECG monitoring system with the benefits of low power and high data transmitted function. Wang used a micro control unit (MCU) to realize the adjustable radio frequency (RF) and power reduction that optimized the ECG system. Another novel technical design is the antenna for wireless devices. Chiang et al. [11] proposed a multiband and power efficiency antenna for a USB dongle application.

MI can be detected by the abnormalities waveform features of the ECG, including ST displacement, T wave inversion, silent Q wave, and so on [1,12]. Hence, it is effective to make use of machine learning algorithms to achieve an automated MI diagnosis [13,14,15]. In the past few years, deep learning (DL) methods, including convolutional neural networks (CNN), recurrent neural networks, restricted Boltzmann machines [16], autoencoder, and generative adversarial networks are proposed [17,18,19,20]. These network architectures or learning methods are used for ECG classification, denoising, reconstruction, annotation, data compression, data generation, and data synthesis purposes. Among all DL methods described above, CNN is the most commonly used method for MI detecting and locating [21]. Acharya et al. [22] proposed a CNN model for MI detection on noisy lead II ECG and reached an accuracy of 93.53%. Alghamdi et al. [23], on the other hand, treated the lead II ECG as a 2D image and employed the transfer learning method by utilizing a pre-trained visual geometry group network (VGG-Net) [24] as a feature extractor incorporating an additional trainable classifier where the yielded accuracy after model retraining was as high as 99.22%. However, the above methods only make use of single-lead ECG information. To this end, Baloglu et al. [25] implemented a simple approach where a single CNN model was trained using all lead signals, resulting in a model that is capable of detecting and locating MI regardless of the input lead. Another way is to regard 12-lead ECG as a 12-by-signal-length image and perform the convolution along the signal direction but not lead direction [26]. A limitation of this is that the features in each lead will be extracted by the same filters. To extract features in each lead independently, Lodhi et al. [27] trained a CNN model for each lead. The 12 models were then used for a voting mechanism to yield a unified prediction of MI appearance, yet such a high number of CNNs will not be practical for portable devices. To improve on this drawback, Reasat et al. [28] proposed a high-level architecture CNN model named shallow-CNN, where each lead was passed through an Inception module [29] to execute feature extraction. The features were then concatenated and performed classification. This way, each lead of features can be extracted independently while giving a single prediction. As those leads are more relevant to inferior MI (IMI), II, III and augmented vector foot (aVF) were utilized to output the prediction between healthy control (HC) and IMI. Liu et al. [30] came up with the same approach, but with a total of six diagnosis classes and using all 12-lead ECG compared to previously mentioned work. The classes include the anterolateral (ALMI), anterior (AMI), anteroseptal (ASMI), inferolateral (ILMI), IMI, and HC, and they called the proposed model MFB-CNN. Han et al. [31] also implemented a similar approach and experiment but with some structural improvements in their ML-ResNet, which includes the residual network (ResNet) to improve the gradient vanishing problem [32] and the batch normalization (BN) to reduce the internal covariant shift [33]. Last but not the least, Hao et al. [34] worked on a similar approach as well but using DenseNet [35], a novel network architecture. Although both ResNet [36] and DenseNet [37] are famous for their skip connection approach, the latter achieved better performance via feature concatenate, whereas the former adopted feature addition.

Among all aforementioned state-of-the-art methods, different issues appear in different studies. In [22,23,24], only single lead information was utilized, which is not sufficient if the multiple MI location predictions are applied. In [25,26], every lead was used together as an input into CNN while training, but the features should be extracted separately. In [27], 12 models were trained, one for each lead and the final prediction was considered using a voting mechanism. However, the fact is that the multi-lead features-concatenate technique used to accomplish extracting inter-lead ECG features independently can be utilized while getting a unified prediction [28,29,30,31,32,34]. While there are plenty of studies that achieve remarkable accuracy [22,23,24,25,26,34], none mentioned and considered intra-individual variability (AIV) nor inter-individual variability (RIV) in ECG [38]. AIV is defined as the variability between ECGs from the same individual or variability within one ECG, while RIV is the variability between ECGs from different individuals. Though not explicitly mentioned in [22,23,24,25,26,34], their experimental scheme can be referred to as a beat-to-beat AIV cross-validation (bAIV-CV) scheme. In [28,29,30,31], the RIV problem was taken into account through implementing RIV cross-validation (RIV-CV), which regards the data from the same individual as a whole to prevent ECGs from appearing in the different folds at the same time. bAIV-CV, on the other hand, simply randomly crop segments from an ECG to form different folds. Even though there may be bAIV contained in the ECG from various sources [38], most of the aforementioned studies have already shown that it is insignificant in the Physikalisch-Technische Bundesanstalt (PTB) database. Simonson et al. [39] showed that AIV is smaller than RIV in ECG. Another potential problem is the so-called class imbalance problem [40], which refers to the data in a class outnumbered the other class. Such scenarios may induce the models to classify samples as belonging to the majority class simply. It can be observed that, while validating MI detection performance, studies that were previously considered and mentioned earlier tend to allow the number of data in the class MI to be significantly larger than in HC. Finally, in [28,29,30,31], features in every lead were utilized independently, but the performance, especially in MI locating, has room for improvement. In machine learning, overfitting is a production of an analysis that corresponds to how close or how exact something is to a particular set of data and may, therefore, fail to fit the held-out data or predict future observations reliably. Overfitting is caused by exceeding some optimal network size, whereas overtraining refers to the excessive time for network training. Both may finally result in losing the predictive ability of the model [41]. Most of the aforementioned literature prevent overtraining by using an early stopping mechanism, which stops the training by monitoring the accuracy or loss. However, none of them take the overfitting problem into account in the process of designing and validating a model. To verify the occurrence of overfitting in the model development phase, one should start from the simplest architecture then gradually increase the complexity when observing the validated result [42]. A. Kumar et al. [43] used a novel fuzzy cross-entropy loss together with transfer learning to increase the accuracy of the model. The work from M. Piekarski et al. [44] mainly focused on the implementation of transfer learning by utilizing various pre-trained networks where the VGG16 outperformed among all models considered in the study. Toward this end, a network should be simple and adjustable where the sequence of double-convolution-follow-by-pooling structure observed in VGG13 [24], together with multiscale features concatenate structure, was inspired by [45,46]. A multi-lead features-concatenate and multiscale features-concatenate integrating structure were developed, which is much like a transposed version of the VGG13 structure. Signals will first be pooled then passed to the convolutional layers to extract features that exist in different scales. The number of inputting scales and the number of filters remain adjustable to find the best architecture and justify overfitting.

**Figure 1:**
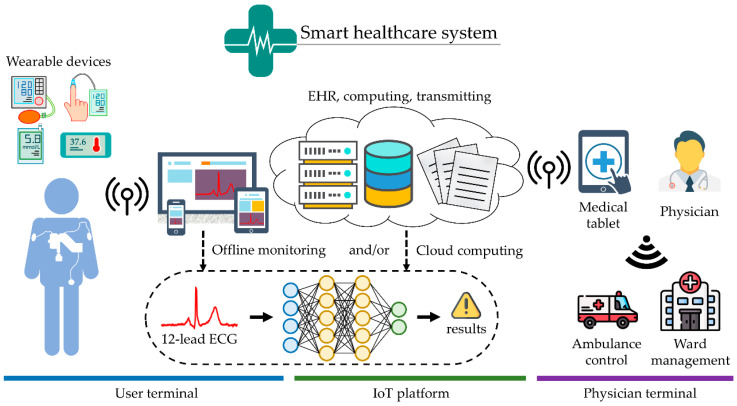
The schematic diagram of healthcare application with integration of Internet of Things (IoT) technology. 12-lead electrocardiogram (ECG) information can combine with other physiological signal values from different wearable devices, such as blood glucose, body temperature, and blood pressure, for algorithm models. Physicians can investigate or diagnosis and undertake further action, such as calling an ambulance or ward, through offline monitoring and/or cloud computing.

As technology advances, utilization of smaller computers together with faster internet speeds for smart devices makes the improvement in portable devices and the Internet of Things nowadays an advanced research area. These open a new and wide range of solutions in e-health, surveillance, and monitoring for medical purposes. However, portable devices are prone to having a lower level of computational capability as well as having a battery consumption problems. This research aims to demonstrate the solutions to address the research challenges in the development of MI detection models using the CNN algorithm to apply feature extraction, classification, and further detection for portable devices. Figure 1 is the schematic diagram of healthcare application related to the research, 12-lead ECG signals, or other physiological signal values captured by portable devices sent to a smart device. It can either choose offline monitoring or cloud computing if the internet is available. Physicians can investigate or diagnose and undertake further actions, such as calling ambulance control or ward management. In other words, the proposed models are kept in both smart devices and cloud servers. Once an abnormality on the ECG waveform is identified by the model, the ECG will be sent to the physician in the hospital for further investigation. A smaller yet more accurate model allows the whole system to be more sustainable and makes it possible to fit inside a smart device for offline monitoring. Since bAIV about the MI and HC is negligible in the PTB database and assessing the RIV-CV has more clinical significance, only RIV-CV will be implemented. Moreover, considering it may lead to model performance reduction, the class imbalance problem should be considered. The main contributions in this research are as follows: First, by carefully verify the occurrence of underfitting and overfitting, the proposed N-Net for MI detection task outperformed the previous works considered in this study. Second, the proposed MSN-Net outperformed both the N-Net and the previous works, which indicates that multiscale features can improve the performance of the model. Third, networks were trained with a different number of input signal scales and a different number of filters in the convolutional layer. The ones that achieved the best accuracy in MI detecting and MI locating were then compared with several state-of-the-art techniques. The comparison was conducted using several parameters, including the nonparametric Mann–Whitney *U* test, to determine the difference of performances between the previous works.

## 2. Materials and Methods

**Figure 2:**
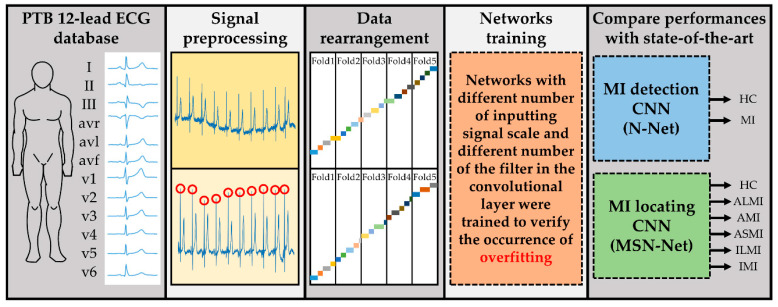
Block diagram of the research, including Physikalisch-Technische Bundesanstalt (PTB), 12-lead ECG database gathering, signal preprocessing, data rearrangement, study model networks training, and performance, compared with other studies. The final objective was to build models capable of detecting and/or locating the occurrence of MI by analyzing the 12-lead ECG.

In this section, the experimental dataset and the signal preprocessing methods are first introduced, including signal denoising and data rearrangement. Next, the network architecture is presented and illustrated. Finally, verifying the performance of the models proposed in this research is discussed. Figure 2 demonstrates the block diagram of the research. The final objective is to build models that are capable of detecting and/or locating the occurrence of MI by analyzing the 12-lead ECG. First of all, the PTB 12-lead ECG databases were gathered, and signals were then denoised and segmented. When forming folders for 5-fold cross-validation, it was necessary to prevent patients’ data from appearing in the different folders at the same time. Next, networks with a different number of inputting signal scales and a different number of filters in the convolutional layer were trained. The ones that achieved the best accuracy in MI detection and/or locating were then compared with several state-of-the-art networks. The comparison was conducted using several clinical indexes, including accuracy (ACC), sensitivity (SEN), specificity (SPE), and F1-score (F1), and area under curve (AUC), together with the nonparametric Mann–Whitney *U* test to determine whether the difference in performances between the previous works and the proposed networks are significant. The training was conducted on several computers with different hardware configurations. Yet, only the version of TensorFlow affected the network performance. In this study, the TensorFlow 2.0.0 GPU version was chosen.

**Figure 3:**
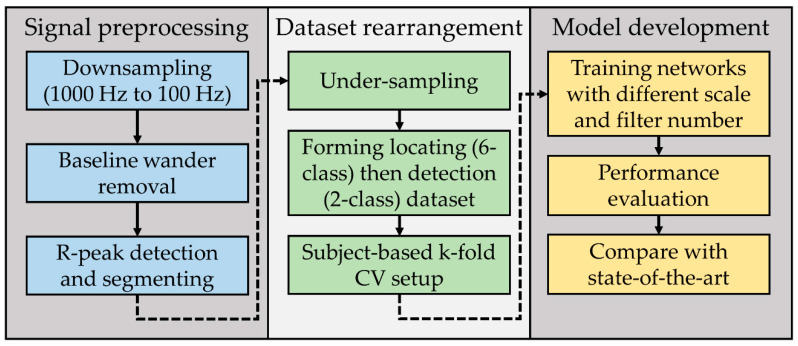
Block diagram of the proposed method, including signal preprocessing, dataset rearrangement, and model development.

Figure 3 is the block diagram of the method used in this research. During the signal preprocessing phase, the signal was down sampled to 100 Hz to reduce the computational cost of the subsequent steps. Next, segmentation using R-peak detection was conducted to shorten the length of the signals, thus simplifying the input layer of the neural network. Afterward, in the dataset rearrangement phase, under sampling was preferred to preliminary tackle the class imbalance issue since there was enough data for all classes. One of the main aims of the proposed research was to compare MI detection and locating performance of the model with previous works. With that, the MI locating dataset was created, and part of it was used to form the MI detection dataset. The k-fold CV was used to provide a measure of how accurately the model could predict across the whole dataset. To fulfill the inter-patient experimental scheme, it was necessary to prevent the patients’ data from appearing in the different folds at the same time. Finally, in the model development phase, instead of testing the performance of one or more fixed network architecture, testing started from a simple network structure and gradually increased the complexity to find the maximum performance between underfitting and overfitting. The adjustable parameters included the number of inputting signal scales and the number of filters in the convolutional layers. The configuration that achieved the best ACC evaluated by k-fold CV was chosen and was compared with several state-of-the-art techniques by some clinical indexes, including ACC, SEN, SPE, F1, and AUC. The proposed networks showed promising results that achieved both higher average performance and significant differences evaluated by the *U* test.

### 2.1. ECG Dataset

**Figure 4:**
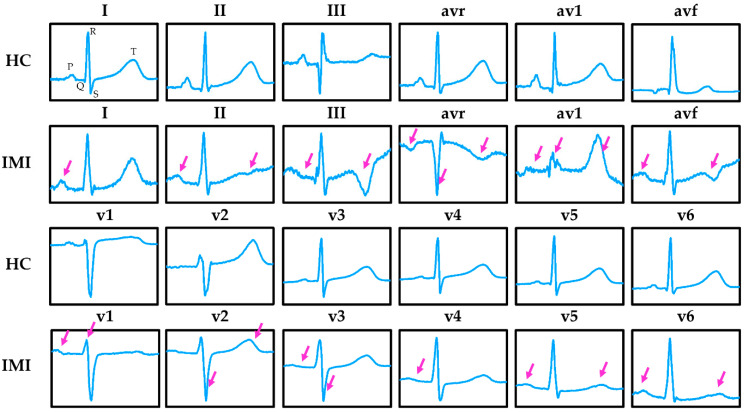
Samples of healthy control (HC) and inferior myocardial infarction (IMI) 12-lead ECG. IMI can be detected by the abnormalities waveform features of the ECG, as indicated by the arrows that include the ST displacement, T wave inversion, silent Q wave, and so on.

To evaluate the performance of the proposed model, the open-access PTB ECG database [47] collected from PhysioNet [48] was used. The PTB ECG database comprises 52 healthy controls (HC) and 148 MI patients. Since patients may have more than one record, a total of 80 HC records and 368 MI records were found. Each record contains a standard 12-lead ECG together with 3 Frank lead ECGs under 1000 Hz sampling rate and 16-bit resolution ranging from −16,384 μV to 16,384 μV. Figure 4 shows the snapshot of the 12-lead HC and IMI ECG. According to the information provided by the header file in the database, MI records can be further divided by occurred MI locating. Five MI locations were utilized in this study, namely 43 ALMI, 47 AMI, 77 ASMI, 56 ILMI, 89 IMI.

### 2.2. Signal Preprocessing

Since the ECG maximum frequency band is about 40 Hz [49], applying an anti-aliasing filter and down sampling to 100 Hz can eliminate high-frequency noise and reduce the computational cost on the later steps of the experiment while still taking the Nyquist theorem into consideration. A median filter with a 0.857-s window was then employed to find and remove baseline wander. The window size was calculated based on the objective of completely covering one cardiac cycle (PQRST waves) under a 70 beats per minute assumption. Denoised signals were then segmented into pieces using the R-peak detection algorithm proposed by Christov [50], while serving as a data augmentation method where every piece of data contained 50 points before the R-peak and 349 points after the R-peak. Lastly, to prevent the model from tending to predict classes that have more training data [40], the quantity of data in each class was required to be equal. This was achieved through random under sampling, a data level solution for imbalanced data [51]. Thirty (30) data pieces were chosen from every 40 chosen records in all 6 diagnostic classes, resulting in a total of 7200 data pieces in the MI locating dataset. Cross-validation (CV) was implemented after model training. A similar method to a CV is called leave-one-out, while the standard method is the leave-one-patient-out CV [52]. However, it was impractical to execute CV on a per subject basis since given a total of 96 chosen patients, 96-fold CV has to be performed. Thence, a subject-based k-fold CV was more suitable for the current investigation. A subject-based 5-fold CV was performed to evaluate the model performance, in which every fold contained multiple patient records. In the meantime, it was necessary to prevent a patient record from appearing in other folds. As for the MI detection dataset, 6 out of 30 were selected from each segmented data in all five MIs to fulfill class balancing. Table 1 summarizes the dataset after the rearrangement.

### 2.3. Network Architecture

**Figure 5:**
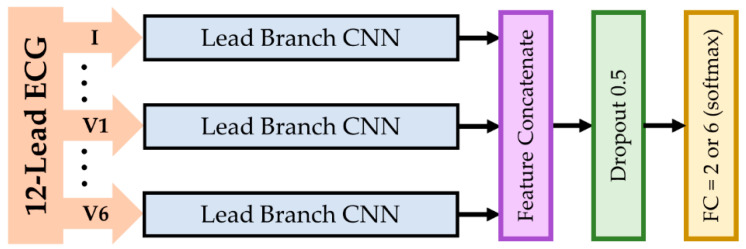
Multi-lead features-concatenate network. Twelve (12) lead branch convolutional neural networks (CNNs) were used to extract features in each lead independently and were then concatenated and classified.

**Figure 6:**
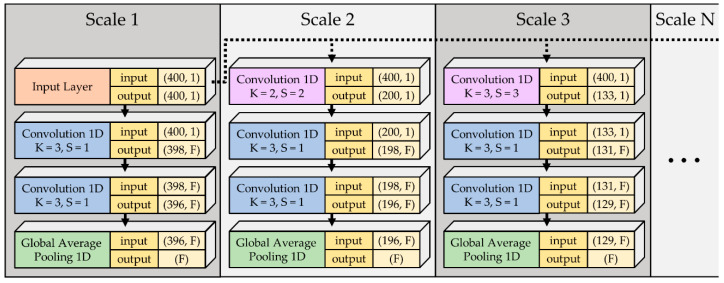
Multi-scale features-concatenate network in every lead branch. K represents kernel size, S represents strides. The N-Net only contained structures illustrated in scale 1, and MSN-Net that used two scales will contain the structure of both scale 1 and scale 2. MSN-Net that used three scales contained structures of scale 1, scale 2 and scale 3, and so on.

First of all, the network took 12-lead ECG at the input layer, as shown in Figure 5. Each lead was fed into a lead branch CNN to extract features independently. In the lead branch CNN, the input was passed through several parallel paths, as illustrated in Figure 6. In each parallel path, the input was sequentially passed through two convolutional layers marked in blue in Figure 6 for features extraction and a global average pooling (GAP) layer for overfitting prevention. To extract larger features, pooling the signal can let the features become smaller, which allows the filters with the same kernel size to extract it. This is in contrast to having a larger kernel size, which has a higher computational cost. Furthermore, instead of using common maximum pooling or average pooling, convolution with both kernel size (K) and stride (S) with the same value was introduced to accomplish the down sampling operation [53], which is considered a learnable pooling layer. Hence a specialized convolutional layer marked in purple in Figure 6 was used to pool the signal for larger features extraction. The extracted features were then concatenated and linked to a fully connected (FC) layer for classification. This FC layer was the only non-convolutional layer in the proposed model. It used the SoftMax activation function, which is the most commonly used activation function in the output layer of a model for classification tasks. To be more specific, 2 neurons for MI detection and 6 neurons for MI locating at the output FC layer were used. The parameter F represents the number of filters on a scale branch. Since non-zero paddings were executed, the convolutional layers colored in blue in Figure 6 reduced the length of the passing signals by 2 since K was 3. All the convolutional layers used the rectified linear unit [54] as the activation function. Preventing overfitting is the utmost important task. Therefore, a dropout layer [55] was inserted with a rate of 0.5 right before FC. Finally, the number of filters and the number of scales were reserved as variables for tuning the best structure. Models using single-scale features were named narrow net (N-Net), while models using multi-scale features were named multi-scale narrow net (MSN-Net).

### 2.4. Model Training

The models described in Section 2.3 were trained with categorical cross-entropy loss, a common loss function for the multi-class classification task, and Adam optimizer [56] with a 0.001 learning rate. The model parameters updated after every 300 data were inputted. Training stops if it did not get any improvement for 20 continuous epochs or when a maximum of 200 epochs was reached. The accuracy and loss at each epoch were recorded. Additionally, how the number of filters and scales affect the model performance was explored. Performances of models with filter numbers from 1 to 10 and scale numbers from 1 to 5 were recorded. Since the robustness of MI locating models using single-scale features were too weak, the performances with filter number up to 15 were recorded. This resulted in a total of 50 different models for MI detection and 55 for MI locating. Since different starting conditions of model parameters led to different final accuracy, a total of 15 times subject-based 5-fold CV were executed for each model. In other words, one model would have 15 sets of CV results. The models were trained in a fixed order of data input to reduce the inter-model accuracy deviation. To compare the results with the current state-of-the-art models proposed by [24,26,27] were retrained since BN was removed and, more importantly, the dataset was different. All the networks were implemented using Keras [57], a neural network library.

### 2.5. Performance Metrics

Predictions given by subject-based 5-fold CV were gathered to form 15 sets of confusion matrixes for both detection and locating models proposed by [28,30,31] as well as this study. The performances of the models were then evaluated in terms of several clinical indexes, including accuracy (ACC), sensitivity (SEN), specificity (SPE), and F1-score (F1) with their corresponding equation for computation as shown in Equations (1)–(4), respectively. Only ACC was evaluated for MI locating models. All above criteria were calculated based on true positive (TP) rate, true negative (TN) rate, false positive (FP) rate, and false negative (FN) rate.

(1) $$\mathrm{ACC}\%=\frac{\mathrm{TP}+\mathrm{TN}}{\mathrm{TP}+\mathrm{TN}+\mathrm{FP}+\mathrm{FN}}\times 100$$

(2) $$\mathrm{SEN}\%=\frac{\mathrm{TP}}{\mathrm{TP}+\mathrm{FN}}\times 100$$

(3) $$\mathrm{SPE}\%=\frac{\mathrm{TN}}{\mathrm{TN}+\mathrm{FP}}\times 100$$

(4) $$F1\%=\frac{2\times \mathrm{TP}}{2\times \mathrm{TP}+\mathrm{FP}+\mathrm{FN}}\times 100$$

In addition to the aforementioned indexes, the receiver operating characteristic (ROC) curve [58] and area under the ROC curve (AUC) of MI detection models were also calculated. As described in Section 2.4, the subject-based 5-fold CV 15 times was performed to evaluate the overall model performances. To verify whether a model had better performance than the other, aside from observing the box plot for comparing the distribution, statistical analysis was also included to determine whether there were significant differences between the performance of the models. Instead of using a *t*-test, the nonparametric Mann–Whitney *U* test [59], also known as the Wilcoxon rank–sum test, was utilized since it is more suitable for small population data. Finally, the AUC and *U* test were calculated using SPSS, a common statistical analysis tool.

## 3. Results

The data preprocessing and rearrangement were executed. The studies in [28,30,31] and the proposed networks in this study were implemented. Subject-based 5-fold and 10-fold CV was performed 15 times for each model in a 2-class detective dataset. Subject-based 5-fold was performed 15 times for each model in a 6-class detective dataset. Before picking the models that achieved the best average accuracy in MI detection or MI locating and compared them with the state-of-the-art, the training and validation accuracies, as well as the loss, were plotted to observe the training effectiveness. Afterward, the comparison was done by determining the significant difference in accuracy using the *U* test. The significant differences in SEN, SPE, F1, and AUC carried out by MI detection models were also calculated and compared.

### 3.1. Accuracy of N-Net and MSN-Net for MI Detection in 2-Class Dataset

**Figure 7:**
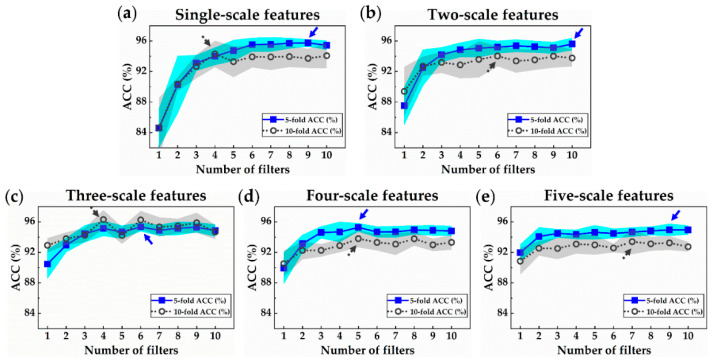
Accuracy trends of the proposed myocardial infarction (MI) detection models plotted in shaded error bar. Blue- and gray-shaded areas indicate the range of one standard deviation, and the best average accuracy under the same scale number is marked by an arrow: Accuracy of models using (**a**) single-scale features; (**b**) two-scale features; (**c**) three-scale features, (**d**) four-scale feature, and (**e**) five-scale features.

Figure 7 shows the ACC trend of the proposed MI detection models with varying filter numbers and scale numbers. It can be observed that at low filter numbers, as the number of filters increased, the ACC increased significantly and gradually converges at about 95% for larger filter numbers. In addition, when using a low filter number, the ACC increased as the number of the inputted signal scale increased. But the increase was not as much as the filter number increase. By observing all the graphs, it can be seen that no matter how complex the network is, the ACC converged to about 95% with a small deviation. In using single-scale features, the best average ACC% of 95.76% was achieved with nine filters by 5-fold validation, and 94.30% was achieved with four filters by 10-fold validation. In using two-scale features, the best average ACC% of 95.60% was achieved with 10 filters by 5-fold validation, and 94.03% was achieved with six filters by 10-fold validation. In using three-scale features, the best average ACC% of 95.33% was achieved with six filters by 5-fold validation, and 96.29% was achieved with four filters by 10-fold validation. In using four-scale features, the best average ACC% of 95.27% was achieved with five filters by 5-fold validation, and 93.80% was achieved with five filters by 10-fold validation. In using five-scale features, the best average ACC% of 94.95% was achieved with nine filters by 5-fold validation, and 93.40% was achieved with seven filters by 10-fold validation. Models that were double confirmed by 5-fold or 10-fold validation demonstrated a similar trend of results. To reduce the number of computer calculations, subsequent verifications were conducted by 5-fold validation. To summarize, using the single-scale features together with nine filters yielded the best result among all models that are for the MI detection task. As the number of used scale features increased, the best average ACC gradually decreased. This may be caused by overfitting due to the excessive model strength, which is proven in Section 3.3.

### 3.2. Accuracy of N-Net and MSN-Net for MI Locating in 6-Class Dataset

**Figure 8:**
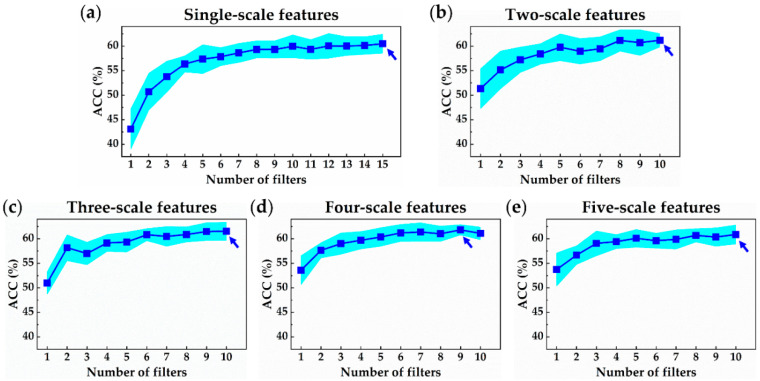
Accuracy trends of our proposed MI locating models plotted in shaded error bar. Blue- and gray-shaded areas indicate the range of one standard deviation and best average accuracy under the same scale number are marked by an arrow: Accuracy of models using (**a**) single-scale features, (**b**) two-scale features, (**c**) three-scale features, (**d**) four-scale features, and (**e**) five-scale features.

Figure 8 shows the ACC trend of the proposed MI locating models with different filter numbers and scale numbers. Similar to MI detection results, it can be observed that at low filter numbers, as the number of filters increased, the ACC increased significantly and gradually converge at about 60% when using larger filter numbers. Furthermore, when using a low filter number, the ACC increased as the number of the inputted signal scale increased. The increase was not as much as the increase in the filter number. By observing all the graphs in Figure 8, it can be seen that no matter how complex the network was, the ACC converged at about 60% with a slightly small deviation. Models that used single-scale features incorporating 15 filters achieved the best average ACC at about 60.49%. Two scales with 10 filters had an ACC of 61.19%, while three scales with 10 filters had an ACC of 61.52%. As for the four scales with nine filters, and five scales with 10 filters, an ACC of 61.82% and 60.87% were achieved, respectively. To summarize, using the four-scale features together with nine filters yielded the best result among all models for the MI locating task. As the number of used scale features increased, the best average ACC increased gradually. This indicates that multi-scale features can help the network in determining the location of MI, which was proved in Section 3.3.

### 3.3. Verifying Training Effectiveness

**Figure 9:**
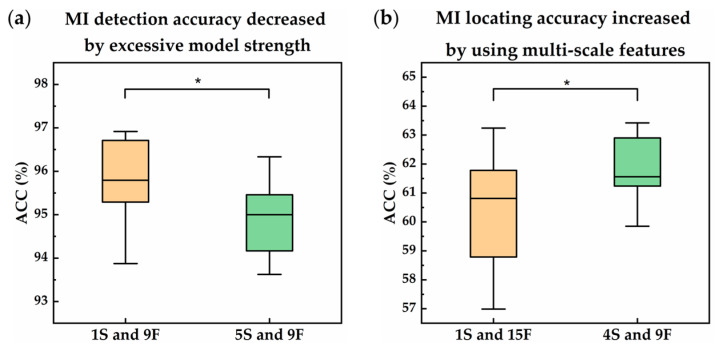
Box plot and significance of the accuracy of the proposed network where 1S indicates models using single-scale features, 9F indicates models using nine filters, and so on. (**a**) Evidence that supports accuracy decrease due to overfitting caused by excessive model strength. The average accuracy decreased from 95.76% to 94.95% (*p* < 0.05); (**b**) Evidence that supports using multi-scale features will increase MI locating accuracy. The average accuracy increased from 60.49% to 61.82% (*p* < 0.05).

To examine the occurrence of overfitting and overtraining, results were collected and analyzed. Figure 9 shows the box plot and significance of the accuracy of the proposed network. It can be observed in the left graph that using a model that was too robust for the current task will, on the contrary, lower the performance of the model from 95.76% to 94.95% (*p* < 0.05) due to overfitting.

**Figure 10:**
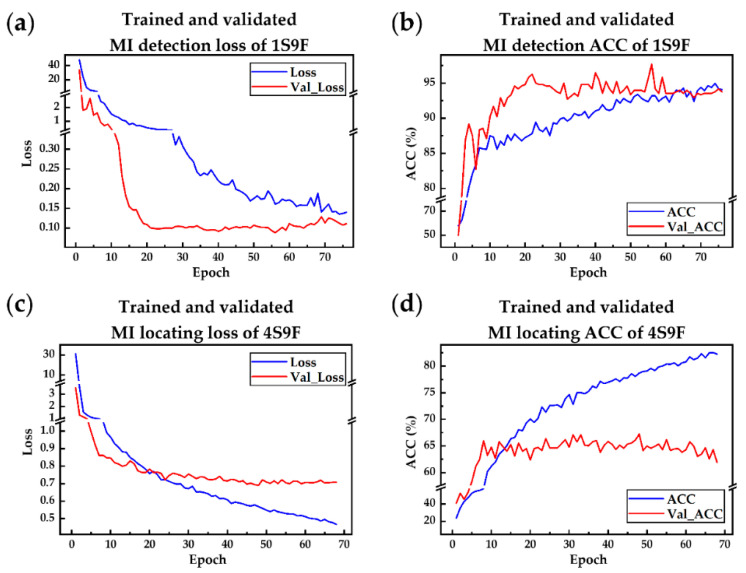
The performances of the proposed networks were recorded during each epoch where 1S indicates networks using single-scale features, 9F indicates networks using nine filters, and so on. (**a**) Trained and validated loss of MI detection model during each epoch; (**b**) Trained and validated the accuracy of MI detection model during each epoch; (**c**) Trained and validated loss of MI locating model during each epoch; (**d**) Trained and validated the accuracy of MI locating model during each epoch.

**Figure 11:**
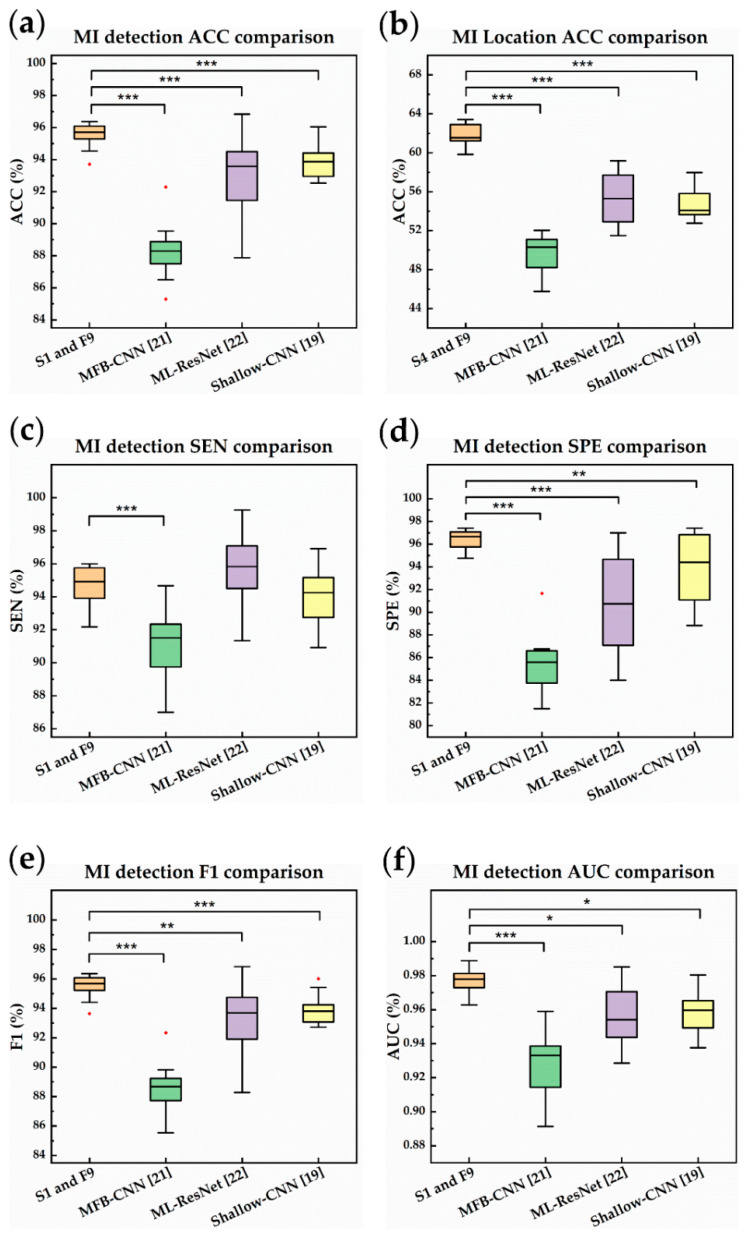
Comparison between the proposed model and literature where 1S indicates models using single-scale features, 9F indicates models using nine filters, and so on. Single star interprets *p* < 0.05, double star interprets *p* < 0.01, and triple star interprets *p* < 0.001. (**a**) MI detection accuracy comparison; (**b**) MI locating accuracy comparison; (**c**) MI detection sensitivity comparison; (**d**) MI detection specificity comparison; (**e**) MI detection F1-score comparison; and (**f**) MI detection area under the receiver operating characteristic curve comparison.

Hence, the use N-Net instead of MSN-Net for MI detection tasks is preferred. On the other hand, using multi-scale features can increase the accuracy of the model from 60.49% to 61.82% (*p* < 0.05) during MI locating tasks. This can be observed in the graph on the right. It is, therefore, useful to use multi-scale features to enhance the performance of the model. In examining the occurrence of overtraining, one should normally be able to determine whether the validated loss was increased during training. However, in this case, both the training and validation curves had a similar trend. It can be found in Figure 10a that both trained and validated loss curves had a similar profile. The loss dropped exponentially and then converged between 0.1 and 0.15. A similar but opposite profile can be found in Figure 11b. Hence, the phenomenon of overtraining did not occur. A significant detail shows that the final trained loss was larger than the final validated loss, whereas the final trained accuracy was higher than validated accuracy. This is due to the fact that the model correctly predicted the label, but only with the confidence that was slightly above 50%. From Figure 10c, it can be seen that during the training stage of the MI locating model, the network had a harsh time optimizing the loss function. Moreover, the validated loss was not as low as that of the trained loss. The same situation can be found in Figure 10d, which points out that there was serious RIV in the dataset, but at least the model was not overtrained. Conclusively, both overfitting and underfitting did not occur in the MI detection model and MI locating model. This is because the models were simple or robust and selected the best one that lies between them. In addition, by examining both the training and validation curves, it can be concluded that overtraining and undertraining were not the case.

### 3.4. Comparing with State-of-the-Art

The MFB-CNN [26], ML-ResNet [31], and Shallow-CNN [28] were retrained under the validating scheme using the rearranged dataset. Fifteen (15) times subject-based 5-fold CV were executed on each model.

The ACC was recorded and used to generate the box plot and calculate the significant difference compared to the proposed model. Only MI detection performances were used to derive SEN, SPE, F1, and AUC, which is due to the fact that these parameters were internally designed to investigate the binary classification performance. By looking at the box plot in Figure 11a,b, it is clearly illustrated that the average accuracy was higher than the rest. By employing the *U* test, *p* < 0.001 was given in all previous works, which implies that the N-Net and MSN-Net outperformed in the MI detection task and MI locating task, respectively. As for clinical indexes shown in Figure 11c,f, the SEN was significantly higher than MFB-CNN (*p* < 0.001). It showed no difference compared to ML-ResNet and Shallow-CNN. The rest of the performances, including SPE, F1, and AUC, showed promising.

## 4. Discussion

N-Net for the MI detection task and an MSN-Net for MI locating task were developed. By carefully designing and validating the network robustness, the performance distribution of N-Net and MSN-Net under different parameters were recorded, and the best one was selected. The results showed that both MI detection and MI locating outperformed the current state-of-the-art. However, the results need to be justified. Furthermore, some detailed issues were not taken into consideration in this work. Additionally, to clarify some potential directions of on-going studies, all the above will be discussed in the following sections.

### 4.1. Verifying the Results of the Proposed N-Net and MSN-Net

In the developing phase of the model, by starting from the simplest network, incrementing the model complexity, and finding the best one among them, the issues of overfitting and underfitting were detected and thus, addressed. In other words, structures that were simpler with respect to the best one can refer to as underfitting and vice versa. Previous works straightforwardly start from a complex model, and as they are revalidated, excessive model strength exhibited a decrease in performances due to overfitting. As such, it is considered that overfitting has also occurred in the network design. Hence, the proposed networks exhibited better performance, and that the proposed networks were less overfitted compared to previous works. During the developing stage of the model, starting from the simplest model and proceeding with gradually increasing the model complexity should be considered.

### 4.2. Issues While Validating the Proposed Network Design

Though the structure of the proposed networks was carefully validated, some detailed issues need to be considered further. First of all, the validation scheme incremented the input signal scale and tested it on each network structure. To improve on this validation scheme, testing can be conducted independently on each kind of input scale. The best ones are then selected and incorporated into those structures and revalidate the new integrated network. Such an experimental scheme can validate which of the input scales contain more features that are more relevant to the task at that particular time, thus utilizing the multi-scale features more effectively. In addition, by comparing the proposed network structure, it can be seen that the design is quite similar to the Inception module [29] and the one that utilizes it on IMI detection [28]. Larger features were extracted by pooling the signals, whereas the latter extract larger features by expanding the kernel size. It remains a question as to which of the network structures would achieve better performance. Another interesting thing to consider is which network can complete one prediction faster. The Inception module [29] extracts larger features by using wider kernels which contain more parameters and will lengthen the time it takes in executing convolution. This directly affects the time required to complete a forward pass, which in turn directly affects the time it takes to give a prediction making it less suitable for real-time portable devices.

### 4.3. Limitations of the Proposed Experimental Scheme

One limitation of the proposed experimental scheme is that the class imbalance problem was addressed using under sampling, a data level method that is simple but makes it impossible to directly compare the results of the proposed model with the other existing works. This is due to the fact that a different dataset in this study was used. To overcome this drawback, a better data level approach can be utilized and can be found in [40] or consider using another category of approaches, called classifier level methods. These classifier level methods keep the dataset unchanged while adjusting the process of learning or training, as discussed in [50].

### 4.4. Potential for Future Study

This study can be extended by developing a computer-aided diagnosis system to lower the computational cost and achieve real-time functionality through a simple yet accurate model. In Section 4.2, examining which of the input scale will yield the best results can be explored. Further validation can be conducted to determine if the proposed design or the Inception modulus [29] is better at extracting multi-scale features. It is also significant to determine and compare the average time it takes for the network to complete a forward pass. Moreover, the geometric separability index [60] can be explored to examine the quality of features extracted by Inception modulus [29] and the proposed method and ultimately, implement the final model in portable devices.

## 5. Conclusions

In this research, data collection, preprocessing, and rearrangement were implemented for training the proposed N-Net and MSN-Net. To carefully verify the occurrence of underfitting and overfitting, two hyperparameters, the number of filters in the convolutional layer, and the number of inputting scales remained variable for tuning the best structure. Furthermore, 15 times patient-based 5-fold CV was run on each candidate model, and the best structures were obtained by comparing the average accuracy. As a result, the N-Net that used single-scale features together with nine filters (1S-9F) yielded a 95.76% average accuracy in MI detection, whereas the MSN-Net that used four-scale features together with nine filters yielded a 60.49% average accuracy in MI locating. By observing indexes including ACC, SEN, SPE, F1, and AUC, together with significant differences evaluated by the *U* test, both N-Net and MSN-Net outperformed the previous works considered in this study in MI detection and locating tasks, respectively, which indicates that previous works encountered the issue of overfitting. Furthermore, the MSN-Net outperformed the N-Net in MI locating tasks, which means that multi-scale features can improve the MI locating performance. More importantly, the proposed networks contained fewer parameters, which points out the potential and suitability in the applications of wearable devices. With all aforementioned promising results, several issues are yet to be explored, such as assessing the quality of the extracted features since it can provide a clearer idea of how to design the network. In future analysis, the testing model can be conducted independently on each kind of input scale. This contains more features relative to the particular time in each task, and then selecting these structures and incorporating them to revalidate the new integrated network to improve the validation. Another recommendation is to deploy the proposed models on portable devices to test their performance on a limited resource device further.

## Figures and Tables

**Table 1:** Patient and data distribution after rearrangement.

Class	No. of Subjects	No. of Records	No. of Pieces (Detection)	No. of Pieces (Locating)
HC	25	40	1200	1200
ALMI	14	40	240	1200
AMI	14	40	240	1200
ASMI	13	40	240	1200
ILMI	16	40	240	1200
IMI	14	40	240	1200

## Data Availability

The PTB ECG database was downloaded from “https://physionet.org/content/ptbdb/1.0.0/, accessed on 9 March 2021”, anyone can access the files, as long as they conform to the terms of the license at “https://physionet.org/content/ptbdb/view-license/1.0.0/, accessed on 9 March 2021”.

## References

[1] Bax J.J., Baumgartner H., Ceconi C. (2012). Third universal definition of myocardial infarction. J. Am. Coll. Cardiol..

[2] Mozaffarian D., Benjamin E.J. (2016). Executive summary: Heart disease and stroke statistics—2016 update: A report from the American Heart Association. Circulation.

[3] Liu B., Liu J. (2015). A novel electrocardiogram parameterization algorithm and its application in myocardial infarction detection. Comput. Biol. Med..

[4] Arif M., Malagore I.A. (2012). Detection and localization of myocardial infarction using k-nearest neighbor classifier. J. Med. Syst..

[5] Wang K., Yang G., Huang Y., Yin Y. (2020). Multi-scale differential feature for ECG biometrics with collective matrix factorization. Pattern Recogn..

[6] Khan M.A., Kim Y. (2021). Cardiac arrhythmia disease classification using LSTM deep learning approach. CMC-Comput. Mater. Con..

[7] Zeng W., Yuan J., Yuan C., Wang Q., Liu F., Wang Y. (2020). Classification of myocardial infarction based on hybrid feature extraction and artificial intelligence tools by adopting tunable-Q wavelet transform (TQWT), variational mode decomposition (VMD) and neural networks. Artif. Intell. Med..

[8] Martis R.J., Acharya U.R. (2014). Current methods in electrocardiogram characterization. Comput. Biol. Med..

[9] Chen Z., Brown E.N. (2010). Characterizing nonlinear heartbeat dynamics within a point process framework. IEEE Trans. Biomed. Eng..

[10] Wang L.-H., Zhang W., Guan M.-H., Jiang S.-Y., Fan M.-H., Abu P.A.R., Chen C.-A., Chen S.-L. (2019). A low-power high-data-transmission multi-lead ECG acquisition sensor system. Sensors.

[11] Chiang W.-Y., Ku C.-H., Chen C.-A., Wang L.-Y., Abu P.A.R., Rao P.-Z., Liu C.-K., Liao C.-H., Chen S.-L. (2019). A power-efficient multiband planar USB dongle antenna for wireless sensor networks. Sensors.

[12] Dohare A.K., Kumar V. (2018). Detection of myocardial infarction in 12 lead ECG using support vector machine. Appl. Soft Comput..

[13] Tripathy R.K., Dandapat S. (2016). Detection of cardiac abnormalities from multilead ECG using multiscale phase alternation features. J. Med. Syst..

[14] Chang P.-C., Lin J.-J., Hsieh J.-C., Weng J. (2012). Myocardial infarction classification with multi-lead ECG using hidden Markov models and Gaussian mixture models. Appl. Soft Comput..

[15] Sadhukhan D., Pal S. (2018). Automated identification of myocardial infarction using harmonic phase distribution pattern of ECG data. IEEE Trans. Instrum. Meas..

[16] Xu S.S., Mak M.W. (2018). Towards end-to-end ECG classification with raw signal extraction and deep neural networks. IEEE J. Biomed. Health Inf..

[17] Wang Y., Song X., Gong G., Li N. (2021). A Multi-Scale Feature Extraction-Based Normalized Attention Neural Network for Image Denoising. Electronics.

[18] Khan M.A., Kim J. (2020). Toward Developing Efficient Conv-AE-Based Intrusion Detection System Using Heterogeneous Dataset. Electronics.

[19] Tor H.T., Ooi C.P., Lim-Ashworth N.S., Wei J.K.E., Jahmunah V., Oh S.L., Acharya U.R., Fung S.S. (2021). Automated detection of conduct disorder and attention deficit hyperactivity disorder using decomposition and nonlinear techniques with EEG signals. Comput. Meth. Prog. Bio..

[20] Ramesh G., Satyanarayana D., Sailaja M. (2020). Composite feature vector based cardiac arrhythmia classification using convolutional neural networks. J. Ambient. Intell. Human. Comput..

[21] Hong S., Zhou Y. (2020). Opportunities and challenges of deep learning methods for electrocardiogram data: A systematic review. Comput. Biol. Med..

[22] Acharya U.R., Fujita H. (2017). Application of deep convolutional neural network for automated detection of myocardial infarction using ECG signals. Inf. Sci..

[23] Detection of Myocardial Infarction Based on Novel Deep transfer Learning Methods for Urban Healthcare in Smart Cities. https://arxiv.org/abs/1906.09358.

[24] Very Deep Convolutional Networks for Large-Scale Image Recognition. https://arxiv.org/abs/1409.1556.

[25] Baloglu U.B., Talo M. (2019). Classification of myocardial infarction with multi-lead ECG signals and deep CNN. Pattern Recognit. Lett..

[26] Liu W., Zhang M. (2017). Real-time multilead convolutional neural network for myocardial infarction detection. IEEE J. Biomed. Health Inf..

[27] Lodhi A.M., Qureshi A.N., Sharif U. A novel approach using voting from ECG leads to detect myocardial infarction. Proceedings of the SAI Intelligent Systems Conference, Center for Healthcare Modeling & Informatics.

[28] Reasat T., Shahnaz C. Detection of inferior myocardial infarction using shallow convolutional neural networks. Proceedings of the 2017 IEEE Region 10 Humanitarian Technology Conference (R10-HTC).

[29] Szegedy C., Liu W. Going deeper with convolutions. Proceedings of the IEEE Conference on Computer Vision and Pattern Recognition.

[30] Liu W., Huang Q. (2018). Multiple-feature-branch convolutional neural network for myocardial infarction diagnosis using electrocardiogram. Biomed. Signal Process.

[31] Han C., Shi L. (2020). ML–ResNet: A novel network to detect and locate myocardial infarction using 12 leads ECG. Comput. Methods Programs Biomed..

[32] He K., Zhang X., Ren S. Deep residual learning for image recognition. Proceedings of the IEEE Conference on Computer Vision and Pattern Recognition.

[33] Ioffe S., Szegedy C. (2015). Batch normalization: Accelerating deep network training by reducing internal covariate shift. IEEE ICMLA.

[34] Hao P., Gao X. (2020). Multi-branch fusion network for myocardial infarction screening from 12-lead ECG images. Comput. Methods Programs Biomed..

[35] Huang G., Liu Z., Van Der Maaten L. Densely connected convolutional networks. Proceedings of the IEEE Conference on Computer Vision and Pattern Recognition.

[36] Xi Z., Niu Y., Chen J., Kan X., Liu H. (2021). Facial Expression Recognition of Industrial Internet of Things by Parallel Neural Networks Combining Texture Features. IEEE Trans. Ind. Inf..

[37] Xie J., He N., Fang L., Ghamisi P. (2021). Multiscale Densely-Connected Fusion Networks for Hyperspectral Images Classification. IEEE Trans. Circ. Syst. Vid..

[38] Schijvenaars B.J.A., van Herpen G. (2008). Intraindividual variability in electrocardiograms. J. Electrocardiol..

[39] Simonson E., Brozek J. (1949). Variability of the electrocardiogram in normal young men. Am. Heart J..

[40] Prati R.C., Batista G.E. Data mining with imbalanced class distributions: Concepts and methods. Proceedings of the Indian International Conference on Artificial Intelligence (IICAI).

[41] Tetko I.V., Livingstone D.J. (1995). Neural network studies. 1. Comparison of overfitting and overtraining. J. Chem. Inf. Model..

[42] Chicco D. (2017). Ten quick tips for machine learning in computational biology. BioData Min..

[43] Kumar A., Vashishtha G., Gandhi C., Zhou Y., Glowacz A., Xiang J. (2021). Novel Convolutional Neural Network (NCNN) for the Diagnosis of Bearing Defects in Rotary Machinery. IEEE Trans. Instrum. Meas..

[44] Piekarski M., Jaworek-Korjakowska J., Wawrzyniak A.I., Gorgon M. (2020). Convolutional neural network architecture for beam instabilities identification in Synchrotron Radiation Systems as an anomaly detection problem. Measurement.

[45] Cui Z., Chen W. (2016). Multi-scale convolutional neural networks for time series classification. IEEE Access.

[46] Jiang G., He H. (2018). Multiscale convolutional neural networks for fault diagnosis of wind turbine gearbox. IEEE Trans. Ind. Electron..

[47] Bousseljot R., Kreiseler D. (1995). Nutzung der EKG-Signaldatenbank CARDIODAT der PTB über das Internet. Biomed. Tech..

[48] Goldberger A.L., Amaral L.A. (2000). PhysioBank, PhysioToolkit, and PhysioNet: Components of a new research resource for complex physiologic signals. Circulation.

[49] Thakor N.V., Webster J.G. (1984). Estimation of QRS complex power spectra for design of a QRS filter. IEEE Trans. Biomed. Eng..

[50] Christov I.I. (2004). Real time electrocardiogram QRS detection using combined adaptive threshold. Biomed. Eng. Online.

[51] Buda M., Maki A. (2018). A systematic study of the class imbalance problem in convolutional neural networks. Neural Netw..

[52] Subject Cross Validation in Human Activity Recognition. https://arxiv.org/abs/1904.02666.

[53] Striving for Simplicity: The All Convolutional Net. https://arxiv.org/abs/1412.6806.

[54] Nair V., Hinton G.E. Rectified linear units improve restricted boltzmann machines. Proceedings of the International Conference on Machine Learning (ICML).

[55] Srivastava N., Hinton G. (2014). Dropout: A simple way to prevent neural networks from overfitting. J. Mach. Learn Res..

[56] A Method for Stochastic Optimization. https://arxiv.org/abs/1412.6980.

[57] Ketkar N. (2017). Introduction to keras. Deep Learning with Python.

[58] Spackman K.A. Signal detection theory: Valuable tools for evaluating inductive learning. Proceedings of the Sixth International Workshop on Machine Learning.

[59] Mann H.B., Whitney D.R. (1947). On a test of whether one of two random variables is stochastically larger than the other. Ann. Math. Stat..

[60] Thornton C. Separability is a learner’s best friend. Proceedings of the 4th Neural Computation and Psychology Workshop.

